# Guardians of Sensation: Evaluating Metformin's Power Against Chemotherapy-Induced Neuropathy

**DOI:** 10.1155/ijbc/2302217

**Published:** 2025-08-20

**Authors:** Hamid Nasrolahi, Ahmad Mosalaei, Susan Andalibi, Shapour Omidvari, Mansour Ansari, Mohammad Mohammadianpanah, Niloofar Ahmadloo, Samineh Sadeghian, Afshin Karimzadeh, Ehsan Mohammad Hosseini

**Affiliations:** ^1^Department of Radiation Oncology, Namazi Hospital, Shiraz University of Medical Sciences, Shiraz, Iran; ^2^Physical Medicine and Rehabilitation Research Center, Shahid Beheshti University of Medical Sciences, Tehran, Iran; ^3^Department of Neurosurgery, Namazi Hospital, Shiraz University of Medical Sciences, Shiraz, Iran

**Keywords:** chemotherapy-induced peripheral neuropathy, CIPN, metformin, nerve conduction studies, neuroprotection, paclitaxel

## Abstract

**Background:** Chemotherapy-induced peripheral neuropathy (CIPN) is a common and debilitating complication of cancer treatment, particularly with agents like paclitaxel. Effective preventive measures for CIPN are limited. Metformin, an antihyperglycemic agent with neuroprotective properties, has shown promise in preclinical studies; however, its clinical utility in preventing CIPN remains underexplored.

**Objective:** This study evaluates the preventive effects of metformin on paclitaxel-induced peripheral neuropathy in breast cancer patients.

**Methods:** A randomized, controlled study was conducted involving 60 breast cancer patients receiving paclitaxel chemotherapy. Patients were assigned to an intervention group receiving metformin (500 mg twice daily) or a control group without metformin. Peripheral nerve function was assessed using nerve conduction studies (NCSs), measuring sensory nerve action potential (SNAP) amplitude, compound muscle action potential (CMAP) amplitude, and distal latency (DL). Clinical neurological symptoms and adverse effects of metformin were monitored throughout the study.

**Results:** Of the 60 enrolled patients, 47 completed the study (26 control and 21 intervention). The incidence of CIPN was lower in the metformin group compared to the control group, although this difference did not reach statistical significance. Metformin was well-tolerated, with mild gastrointestinal side effects being the most common adverse events. No significant differences between the groups were observed in SNAP amplitude, CMAP amplitude, or DL.

**Conclusion:** Metformin may modestly reduce the incidence of CIPN in patients receiving paclitaxel chemotherapy, although the observed effect was not statistically significant. Given its safety profile and potential neuroprotective benefits, metformin warrants further investigation in larger, multicenter trials to confirm its role in CIPN prevention.

## 1. Introduction

Chemotherapy remains a cornerstone in the treatment of cancer; however, its effects on normal cells and tissues can lead to a range of complications, significantly impacting patient quality of life. Among these, chemotherapy-induced peripheral neuropathy (CIPN) is one of the most prevalent and distressing side effects, often necessitating dose reduction or treatment interruption, thereby compromising therapeutic efficacy [1, 2]. The reported prevalence of CIPN varies widely, affecting approximately 19%–85% of patients undergoing chemotherapy, depending on the agent used and individual patient factors [3].

The primary symptoms of CIPN include pain, paresthesia, numbness, and hypersensitivity. Patients frequently report numbness and tingling, which are often debilitating [4]. Unfortunately, current strategies for preventing or managing CIPN are limited, with treatment options primarily focused on symptomatic relief rather than prevention or reversal of the underlying damage. Preventing CIPN without reducing the therapeutic dose of chemotherapy remains a significant clinical challenge [5].

CIPN is a dose-dependent complication caused by various chemotherapeutic agents, including cisplatin and paclitaxel. Numerous studies have investigated potential preventive and therapeutic strategies for CIPN, but no standard treatment has yet been established [6, 7]. Therefore, identifying effective interventions to prevent or mitigate CIPN is critical for improving patient outcomes and enhancing quality of life during cancer treatment.

Electrophysiological evaluations, such as nerve conduction studies (NCSs), are invaluable for objectively assessing peripheral nerve function in patients with CIPN. NCS measures the conduction of electrical impulses along motor and sensory nerves, providing quantitative data on nerve function and allowing the characterization of axonal or demyelinating neuropathy [8]. Sensory nerve action potential (SNAP), an electrophysiological measure recorded during sensory NCSs, reflects the summated electrical activity of sensory axons. SNAP amplitude, which represents the magnitude of sensory nerve response, is a sensitive marker of axonal loss or dysfunction [6, 9]. A reduction in SNAP amplitude is commonly observed in CIPN and correlates with clinical symptoms such as numbness and tingling [3].

Similarly, compound muscle action potential (CMAP) is an indicator of motor nerve function and is recorded during motor NCSs. CMAP amplitude represents the strength of the motor response, providing insight into motor axonal integrity. Reduced CMAP amplitude is often associated with axonal damage, which can lead to motor weakness and impaired mobility [7]. Distal latency (DL), another critical parameter measured during NCS, indicates the time taken for an electrical impulse to travel from the stimulation site to the recording site at the distal muscle or sensory nerve. Prolonged DL is often a sign of demyelination or conduction slowing, which are characteristic features in some cases of CIPN [5].

Metformin, a widely used antihyperglycemic agent, has gained attention for its potential neuroprotective effects beyond its role in diabetes management. Metformin's primary mechanism of action involves the activation of adenosine monophosphate-activated protein kinase (AMPK), a key regulator of cellular energy homeostasis [10, 11]. Preclinical studies have demonstrated that metformin can reverse mechanical allodynia and reduce the excitability of sensory nerve cells exposed to growth factors and cytokines, mechanisms implicated in chemotherapy-induced neuropathic pain [12, 13]. Moreover, metformin has been shown to exert neuroprotective effects in various models of neurological injury, including diabetic neurodegeneration, ethanol-induced neuroapoptosis, and ischemic stroke [11, 13].

Despite promising preclinical findings, the potential of metformin to prevent or reduce paclitaxel-induced neuropathy has been insufficiently studied in clinical settings. Given its multifaceted neuroprotective mechanisms and the ability of NCS to provide objective metrics for assessing peripheral nerve function, metformin represents a promising candidate for CIPN prophylaxis. This study is aimed at evaluating the preventive effects of metformin on paclitaxel-induced neuropathy in cancer patients. The findings from this investigation, which include electrophysiological parameters such as SNAP amplitude, CMAP amplitude, and DL, could provide valuable insights for clinicians and inform strategies to mitigate the adverse effects of chemotherapy, ultimately improving patient care and quality of life.

## 2. Methods and Materials

This randomized, controlled study was conducted on 60 breast cancer patients referred to the radio-oncology clinic of Namazi Hospital at Shiraz University of Medical Sciences in 2019. Eligible patients scheduled to receive paclitaxel chemotherapy were randomized into two groups: one receiving metformin and the other serving as a control. The ethics committee of Shiraz University of Medical Sciences approved the study protocol with Reference Number IR.SUMS.MED.REC.1398.318, and the written informed consent was obtained from all participants.

### 2.1. Inclusion and Exclusion Criteria

The inclusion criteria were as follows:

1. Pathologically confirmed breast cancer.
2. Age between 18 and 75 years.
3. Absence of diabetes mellitus, confirmed by fasting blood sugar (FBS) levels below 125 mg/dL and random blood sugar (BS) levels below 140 mg/dL.
4. Normal renal and liver function tests.
5. ECOG (Eastern Cooperative Oncology Group) functional status of 0 or 1, which indicates that patients are relatively well-functioning despite their illness.

Exclusion criteria included the following:

1. History of diabetes mellitus.
2. Use of carbonic anhydrase inhibitor drugs.
3. Presence of neurological or systemic diseases.
4. Risk factors for metformin-related lactic acidosis, such as congestive heart failure (New York Heart Association Class III or IV) or a history of any type of acidosis.
5. Pregnancy.

### 2.2. Study Design and Randomization

Patients were randomly assigned to two groups of 30 patients each. The intervention group received metformin, 500 mg orally twice daily after meals, concurrently with paclitaxel chemotherapy. The control group did not receive metformin or any placebo. Randomization was performed using a simple randomization method.

### 2.3. Baseline Assessments

Patient demographic and clinical characteristics, including age, sex, and medical history, were recorded. A comprehensive physical examination was conducted, and height, weight, and body mass index (BMI) were documented. Fasting blood glucose levels were measured at baseline to confirm the absence of diabetes mellitus and were repeated twice during the treatment period to ensure no development of diabetes.

### 2.4. Neurological Assessments

A four-limbed NCS was conducted by a trained physiatrist at baseline and after completing chemotherapy. NCS evaluated the amplitude and delay in the following nerves:

- Upper extremities: ulnar and radial nerves (sensory) and median nerve (motor).
- Lower extremities: sural nerve (sensory) and tibial and peroneal nerves (motor).

### 2.5. Intervention and Chemotherapy Protocol

Patients in the intervention group received metformin, 500 mg twice daily, starting on the first day of paclitaxel chemotherapy. Paclitaxel was administered intravenously at a dose of 175 mg/m^2^ over 3 h, every 2–3 weeks, for four cycles. The control group received no additional medication during chemotherapy.

### 2.6. Follow-Up and Monitoring

Patients were monitored weekly for signs of neurological symptoms such as numbness, paresthesia, or other sensory abnormalities. They were also assessed for metformin-related side effects, including

- gastrointestinal symptoms (e.g., heartburn, stomach pain, nausea, bloating, diarrhea, and constipation),
- weight changes,
- headache,
- anemia, and
- unpleasant metallic taste in the mouth.

### 2.7. Outcome Measures

The primary outcome was the reduction in CIPN, as assessed by changes in NCS results and clinical neurological symptoms between baseline and the study endpoint. The secondary outcome was the incidence and severity of metformin-related adverse effects. Data were recorded in structured forms during weekly follow-ups and at the final visit.

### 2.8. Statistical Analysis

Data analysis was performed using appropriate statistical tests to compare neuropathy outcomes and side effects between the intervention and control groups. Continuous variables were expressed as mean ± standard deviation, and categorical variables were presented as frequencies or percentages. Statistical significance was defined as a *p* value < 0.05.

## 3. Results

This study initially enrolled 60 patients with breast cancer; however, 47 patients completed the protocol, which included both metformin administration and NCSs. Of these, 26 patients were in the control group, and 21 were in the intervention group.

The mean age of patients in the intervention group was 62.8 years (±6.0), while the mean age in the control group was 60.6 years (±5.4). This difference was not statistically significant (*p* value = 0.911). Nearly all participants were female, with only one male in each group.

The mean BMI was 27.9 (±2.9) in the control group and 28.5 (±3.5) in the intervention group, a difference that was also not statistically significant (*p* value = 0.560).

After completing four cycles of paclitaxel chemotherapy, the mean cumulative dose of paclitaxel was 1205 mg (±119.0) in the intervention group and 1220 mg (±128.2) in the control group. This difference was not statistically significant (*p* value = 0.636).

In the intervention group, the satisfaction rate with metformin treatment was 66.7. The most commonly reported adverse effect was bloating, observed in 26.6% of patients. Other frequently reported side effects included nausea and diarrhea. Anemia and heartburn were reported less frequently. None of these side effects were severe enough to necessitate discontinuation of metformin.

Clinical sensory and motor deficits did not differ significantly between the intervention and control groups. The impact of metformin on the incidence of CIPN was analyzed using a *t*-test. The results indicated a lower risk of CIPN in the intervention group compared to the control group, but this difference was not statistically significant (Table 1).

## 4. Discussion

CIPN remains a significant clinical challenge, particularly for patients receiving neurotoxic agents such as paclitaxel. In recent years, metformin has emerged as a potential neuroprotective agent with promising findings in preclinical and clinical studies. The present study was aimed at evaluating the efficacy of metformin in preventing and mitigating CIPN in a cohort of breast cancer patients treated with paclitaxel.

The results demonstrated a lower incidence of CIPN in the group receiving metformin concurrently with paclitaxel compared to the control group. Although this difference did not reach statistical significance, the relative reduction in neuropathy (16%) suggests a potentially meaningful clinical effect that warrants further investigation in larger studies with greater statistical power.

Several preclinical and clinical studies support the neuroprotective effects of metformin. In a study by Mao-Ying et al., metformin was shown to attenuate mechanical allodynia and prevent sensory deficits in mice treated with cisplatin and paclitaxel. This study highlighted that metformin coadministration reduced tactile hypersensitivity and preserved intraepidermal nerve fiber density in the paws of treated mice, suggesting its capacity to protect peripheral nerves from chemotherapy-induced damage [14].

El-Fatatry et al. conducted a randomized clinical trial to assess metformin's role in mitigating oxaliplatin-induced peripheral neuropathy in colorectal cancer patients. This study reported a significantly lower incidence of Grades 2 and 3 neuropathy in the metformin group (60% vs. 95%; *p* < 0.001). These findings strongly suggest metformin's potential in reducing neurotoxicity across various chemotherapy agents [15].

In the study by Mo et al., lithium and ibudilast were investigated as potential agents for preventing paclitaxel-induced neuropathy. The results demonstrated that pretreatment with these agents reduced the severity of CIPN, allowing higher doses of paclitaxel to be administered. These findings underscore the importance of identifying pharmacological interventions that not only reduce neuropathy but also enable more aggressive chemotherapy regimens to be safely administered [16].

The findings of the present study align with these prior investigations, demonstrating a trend toward reduced neuropathy incidence in the metformin group. While the results did not reach statistical significance, the observed reduction in CIPN incidence is encouraging and indicates the need for future studies with larger sample sizes and optimized protocols to validate these preliminary observations.

This study has several limitations. First, the relatively small sample size and high attrition rate due to noncompliance with the drug regimen may have impacted the statistical power and generalizability of the findings. Second, the short follow-up duration may have precluded the detection of late-onset neuropathy or the long-term benefits of metformin. However, this study is among the first to clinically evaluate metformin's effect on paclitaxel-induced neuropathy, contributing valuable insights to this emerging area of research.

Despite these limitations, the safety profile of metformin observed in this study is noteworthy. Metformin was well-tolerated, with minimal side effects reported, and its use was associated with reduced pain and improved patient satisfaction. Given its well-established safety and affordability, metformin represents an attractive candidate for further investigation in multicenter clinical trials with larger and more diverse patient populations.

In conclusion, this study suggests that metformin has the potential to modestly reduce the incidence of CIPN in patients treated with paclitaxel, with promising implications for its use in supportive cancer care. While additional research is necessary to confirm these findings, the results of this study provide a foundation for future exploration of metformin as a neuroprotective agent in the oncology setting.

## Figures and Tables

**Table 1:** Effect of metformin on the incidence of CINP in patients treated with paclitaxel.

**Nerve**		**Amplitude (** **m** **e** **a** **n** ± **SD)**			**Latency (** **m** **e** **a** **n** ± **SD)**		
		**Case (** **n** = 21**)**	**Control (** **n** = 26**)**	**p** ** value**	**Case (** **n** = 21**)**	**Control (** **n** = 26**)**	**p** ** value**
Sensory (SNAP)	Rt ulnar	21.3 ± 7.1* μ*V	20.6 ± 6.9* μ*V	0.73	2.8 ± 1.1 ms	2.7 ± 0.9 ms	0.73
	Lt ulnar	21.3 ± 6.5* μ*V	18.8 ± 6.5* μ*V	0.19	2.8 ± 1.2 ms	2.6 ± 1.1 ms	0.55
	Rt radial	27.2 ± 7.1* μ*V	28.5 ± 7.5* μ*V	0.54	2.4 ± 1.3 ms	2.4 ± 1.2 ms	0.98
	Lt radial	26.4 ± 6.2* μ*V	24.7 ± 6.8* μ*V	0.38	2.3 ± 1.5 ms	2.4 ± 1.3 ms	0.80
	Rt sural	13.1 ± 5.3* μ*V	12.7 ± 5.8* μ*V	0.80	3.2 ± 1.4 ms	3.6 ± 1.4 ms	0.33
	Lt sural	12.8 ± 5.3* μ*V	14.1 ± 8.4* μ*V	0.54	3.6 ± 1.8 ms	3.5 ± 1.7 ms	0.84

Motor (CMAP)	Rt median	6.5 ± 1.2 mV	6.1 ± 1.4 mV	0.30	3.3 ± 0.7 ms	3.3 ± 0.7 ms	0.56
	Lt median	6.8 ± 1.3 mV	8.7 ± 6.0 mV	0.16	2.8 ± 0.7 ms	3.0 ± 0.7 ms	0.99
	Rt peroneal	4.0 ± 2.0 mV	3.8 ± 1.4 mV	0.68	4.4 ± 1.4 ms	4.5 ± 1.4 ms	0.80
	Lt peroneal	4.4 ± 1.6 mV	4.9 ± 1.8 mV	0.32	4.2 ± 1.1 ms	3.9 ± 1.3 ms	0.40
	Rt tibial	4.8 ± 1.2 mV	5.1 ± 1.0 mV	0.35	4.5 ± 0.8 ms	4.4 ± 0.8 ms	0.67
	Lt tibial	4.9 ± 1.4 mV	4.9 ± 1.1 mV	0.99	3.8 ± 0.9 ms	3.6 ± 1.0 ms	0.48

Abbreviations: *μ*V, microvolts; CMAP, compound muscle action potential; ms, milliseconds; mV, millivolts; SNAP, sensory nerve action potential.

## Data Availability

The raw data are available from the corresponding author upon request.
